# Lung Transplantation for Late-Onset Pulmonary Hypertension in a Patient with Congenital Diaphragmatic Hernia

**DOI:** 10.1055/s-0038-1675377

**Published:** 2018-12-26

**Authors:** Chiara Iacusso, Francesco Morini, Irma Capolupo, Andrea Dotta, Stefania Sgrò, Francesco Parisi, Adriano Carotti, Pietro Bagolan

**Affiliations:** 1 Department of Neonatal Surgery, Bambino Gesù Children's Hospital, Rome, Italy; 2 Department of Anesthesiology, Bambino Gesù Children's Hospital, Rome, Italy; 3 Thoracic Transplant Unit, Bambino Gesù Children's Hospital, Rome, Italy; 4 Division of Pediatric Cardiac Surgery, Department of Pediatric Cardiology and Cardiac Surgery, Bambino Gesù Children's Hospital, Rome, Italy

**Keywords:** congenital diaphragmatic hernia, lung transplantation, heart–lung transplantation, pulmonary hypertension

## Abstract

Lung hypoplasia and pulmonary hypertension (PH) in association with congenital diaphragmatic hernia (CDH) may cause fatal respiratory failure. Lung transplantation (Ltx) may represent an option for CDH-related end-stage pulmonary failure. The aim of this study is to report a patient with CDH who underwent Ltx or combined heart-lung transplantation (H-Ltx). Our patient was born at 33 weeks of gestation, with a prenatally diagnosed isolated left CDH. Twenty-four hours after birth, she underwent surgical repair of a type D defect (according to the CDH Study Group staging system). Postoperative course was unexpectedly uneventful, and she was discharged home at 58 days of life. Echocardiography before discharge was unremarkable. Periodic follow-up revealed gastroesophageal reflux (GER) and initial scoliosis. At the age of 10, she was readmitted for severe PH. Lung function progressively deteriorated, and at the age of 14, she underwent H-Ltx due to end-stage respiratory failure. After discharge, she developed recurrent respiratory tract infections, severe malnutrition, and drug-induced diabetes. Scoliosis and GER progressed, requiring posterior vertebral arthrodesis and antireflux surgery, respectively. Bronchiolitis obliterans further impaired her respiratory function, and though she had a second Ltx, she died at the age of 18, 4 and 1.5 years after the first and the second Ltx, respectively. Late-onset PH is an ominous complication of CDH. From our patient and the six further cases collected from the literature, Ltx may be considered as a last-resource treatment in CDH patients with irreversible and fatal respiratory failure, although its prognosis seems unfair.

## Introduction

Congenital diaphragmatic hernia (CDH) is a congenital anomaly characterized by a defect in the diaphragm, lung hypoplasia, and abnormal vascular remodeling. 1 This condition can lead to pulmonary hypertension (PH) and fatal respiratory failure. The impact of pulmonary vascular disease starts in utero and can also contribute to postnatal systolic and diastolic cardiac dysfunction in patients with CDH. It is well-known that severe PH complicates the course of CDH and is associated with poor survival. Despite medical and surgical advances, PH remains a major problem in CDH patients, leading to progressive deterioration in pulmonary function, impaired oxygenation, and respiratory failure. 2 3

Although PH may present (or relapse) at an older age, little is known about delayed presentation of PH in CDH patients and its treatment, in particular the potential role of lung or heart–lung transplantation in these patients. 3

The aim of this paper is to report a patient with CDH who underwent lung transplantation (Ltx) 15 years after CDH repair for delayed presentation of PH and required heart-lung retransplantation (H-Ltx) at 17 years of age for chronic rejection.

## Case Report

Our patient was born at 33 weeks of gestation by cesarean section to a 36-year-old gravida 3, para 2 woman after a prenatal diagnosis of left CDH (31 weeks' gestation). Postnatal chest X-rays confirmed the left CDH, with the liver in the thorax. Initial echocardiogram revealed only a small patent ductus arteriosus. There were no signs of PH at this time. At 24 hours of life, she underwent surgical repair of a huge diaphragmatic defect (type D according to the CDH Study Group staging system) 4 that required a silastic patch closure. Weaning from mechanical ventilation was achieved on postoperative day (POD) 4, but 3 days later she was reintubated for recurrent respiratory distress. Respiratory support was finally stopped on POD 26, and she was discharged home on POD 58. Predischarge echocardiography was unremarkable. The infancy was uneventful and the quality of life was excellent. She underwent periodic follow-up visits that revealed gastroesophageal reflux (GER) and initial mild scoliosis. At the age of 10, she was admitted for abdominal pain, vomiting, and dyspnea, and GER disease and severe PH were diagnosed. Heart ultrasound showed an estimated systolic right ventricular pressure of 130 mm Hg, with a systolic blood pressure of 100/50 mm Hg. Medical treatment for PH was initiated, including sildenafil, bosentan, and furosemide. Since PH progressively worsened despite maximal medical treatment, leading to end-stage respiratory failure, at the age of 14, she was listed for H-Ltx, and 3 months later she received the new organs. Postoperative course was characterized by short-term complications such as severe central venous line-related infections, massive pleural effusion and ascites, and delayed complications, including CDH recurrence with intestinal obstruction, requiring laparotomy and ileostomy formation. In addition, she suffered from recurrent respiratory infections, severe malnutrition, and drug-induced diabetes, significantly impacting her quality of life. With aging, scoliosis and GER also aggravated, requiring posterior vertebral arthrodesis and Nissen's fundoplication at 16 years of age. At 17 years of age, she developed a further episode of pneumonia-related respiratory failure and required a tracheostomy for multiple failed attempts of extubation. Bronchiolitis obliterans deteriorated her respiratory function, and the same year she was relisted for Ltx and underwent her second bipulmonary transplantation.

Despite the second transplant, her general conditions rapidly deteriorated, she developed chronic lung rejection, and she eventually died at the age of 18, 4 and 1.5 years after the first and the second Ltx, respectively.

## Discussion

We report a patient who underwent H-Ltx and subsequent Ltx for CDH-related end-stage PH.

Along with our patient, eight children with CDH who underwent Ltx or H-Ltx have been described ( Table 1 ). 1 2 3 5 6 Two were reported among 31 cases of children who underwent Ltx for a so-called diffuse lung disease and have very few data available. 6 Therefore, for most data, we will refer to the six with available data. All, except one, had a prenatal diagnosis of CDH and five had left side defect. The CDH repair was performed between 3 and 19 days after birth. Four patients underwent CDH repair during extracorporeal membrane oxygenation (ECMO). A diaphragmatic patch was required in four cases: two underwent primary closure, whereas the modality of closure was not specified in the remaining two patients. One patient had major associated anomalies (double-inlet left ventricle, transposition of the great arteries, subaortic stenosis, coarctation of the aorta). In all patients, early (at birth) or delayed (in adolescence) PH was the indication for Ltx or H-Ltx. Three patients had an H-Ltx, one had a single Ltx and transplant pneumonectomy 3 years later, one had a bilateral Ltx in a single step, one underwent sequential lungs transplantation, and two had unspecified Ltx. In all patients who had ECMO, Ltx was performed before 6 months of age (in the neonatal period in 2), whereas indication to Ltx developed in adolescence in the remaining two. In the early transplantation group, the time in the waiting list for emergency transplantation was 8 to 22 days. Median age at transplant was 105 days (range: 0–17 years). The donor's lung reduction was required in one patient only, and a right middle lobectomy was performed.

**Table 1:** Literature review of CDH patients who underwent lung or heart-lung transplantation

Author	Year	No. of patients	Prenatal diagnosis	ECMO	CDH side	Type of Tx	Age at Tx	Outcome	Follow-up
Van Meurs et al	1994	1	No	Yes	R	Lung	17 d	Alive	4 y
Lee et al	2002	1	18 wk	Yes	L	Lung	36 d	Died 51 d post-Tx	–
Lee et al	2002	2	27 wk	Yes	L	Lung	105 d	Alive	3 y
Lee et al	2002	3	18 wk	Yes	L	Heart–lung	19 d	Died 84 d post-Tx	–
Rama et al	2010	1	ns	ns	ns	Lung	ns	ns	ns
Rama et al	2010	2	ns	ns	ns	Lung	ns	ns	ns
Schmidt et al	2013	1	ns	Yes	L	Lung	10 y	Died 109 d post-Tx	–
Iacusso et al (this study)	2017	1	31 wk	No	L	Heart–lung Lung	12 y 17 y	Died 4 y after first Tx	–

Abbreviations: CDH, congenital diaphragmatic hernia; ECMO, extracorporeal membrane oxygenation; ns, not significant; Tx, transplantation.

Our patient shows the dark side of CDH-associated PH that may develop or recur late after a substantial period of well-being. In CDH patients, PH may persist or recur despite negative echocardiographic findings. In a recent study on pulmonary blood flow and vascular resistance, Zussman et al found that five out of eight patients with higher pulmonary artery pressure and vascular resistance and reduced blood flow as compared with controls did not have signs of PH on their echocardiogram. 7 Late or recurrent PH may represent a significant and severe morbidity in CDH patients. Burgos et al found that two out of seven late mortalities in CDH patients were related to persistent or recurrent PH. 8 In one patient, PH recurred at 9 years of age, following an asymptomatic period of several years free from medications. This patient is similar to our own patient who was well and medication-free up to 10 years of age when a diagnosis of severe PH was made. These data suggest that follow-up of CDH patients should carefully address the issue of persistent/recurring PH, finding a reasonable balance between the aggressiveness of investigations and the risks of missing the diagnosis.

Our case and the few others reported show that Ltx may represent a last-resort, life-prolonging option for end-stage PH in CDH patients, although associated with a final ominous prognosis. The first Ltx in a CDH patient was described by van Meurs et al in 1994. 2 They performed a lobar Ltx in a patient in whom other available strategies had failed. The patient underwent ECMO as a bridge to transplantation, and organ transplantation as a bridge to recovery of normal function in the paired organ. The Ltx was not considered as a definitive therapy because long-term immunosuppression, with its risks, may be avoided if the contralateral lung develops adequately. At 4 years of age, the patient underwent a transplant pneumonectomy for severe side effects of chronic immunosuppression 1 that was tolerated well. No data are available on further follow-up. Lee et al report on three patients with CDH who underwent Ltx at 19, 36, and 105 days of age, respectively. 3 The patient who underwent Ltx at 105 days of age was alive at 3 years of follow-up. Patients who underwent Ltx at a younger age died after 84 and 51 days, respectively, suggesting that age at Ltx may influence survival. It is possible that CDH patients with worse lung function, bad general status, and higher PH required transplant earlier and had poorer prognosis and thus worse outcome. On the other hand, the patient reported by van Meurs et al was transplanted at 19 days of age and was alive for 4 years after Ltx. 2 Therefore, it is not possible from these scant available data to detect any pattern of survival based on the age at transplant. Overall, in this small collected series of 6 CDH patients who underwent Ltx and available data, we found only two survivors. Three died shortly after transplant (one had a brain hemorrhage while on ECMO, one could not be weaned off ECMO, and one due to adenovirus infection waiting for re-Ltx), and our patient died 1.5 years after the second transplant due to chronic lung rejection. Only two patients can be considered as long-term survivors, although data are available only up to 3 and 4 years of follow-up, respectively. These figures seem worse than the data from the overall pediatric Ltx population, where a 5-year survival rate of 50% is reported. 9

In CDH patients, early Ltx was proposed as a bridge in patients with severe PH and hypoplasia, waiting for pulmonary parenchyma to grow. 2 3 However, very few studies address the issue of Ltx in CDH patients. It is possible that Ltx in very young infants is technically more demanding or it may be more complex to obtain donor organs, preventing Ltx in small CDH patients. Accordingly, less than 5% of all pediatric Ltx reported in the Registry of the International Society for Heart and Lung Transplantation between January 2008 and June 2016 were performed in infants aged less than 1 year. 10

In conclusion, our case confirms the possibility of late PH occurrence or recurrence in CDH survivors even after a period of well-being and its ominous prognosis. The literature on the issue of Ltx in CDH patients is very scant and heterogeneous maybe due to the lack of available donor organs for small CDH infants and the rarity of late occurrence/recurrence of PH. Despite this limitation in the evidence, Ltx may be considered as a last-resort therapy for patients with CDH and severe PH unresponsive to conventional treatment, conceding time for the lung to grow and allow adequate oxygenation to the patient.

## Notes

New Insights and the Importance for the Pediatric Surgeon In patients with CDH, persistent/late onset pulmonary hypertension is rare but potentially ominous. Long-term follow-up is necessary. In CDH patients with end-stage respiratory failure due to pulmonary hypertension lung transplantation may be considered as a last resort treatment. Single lung transplantation, followed by transplant pneumonectomy, may represent a bridge to allow contralateral lung development, without the long term effects of immunosuppression.
